# Blood-Based Characterization of Hypoxia-Tolerant and Hypoxia-Susceptible Atlantic Salmon (*Salmo salar*) Under Acute Hypoxic Stress

**DOI:** 10.3390/ani16162582

**Published:** 2026-08-18

**Authors:** Salinas-Parra Nicolás, Caballero Paz, Romero Alfonso, Martínez Karin, Soto Carlos, Gallardo-Matus José

**Affiliations:** 1 Laboratorio de Genética y Genómica Aplicada, Escuela de Ciencias Del Mar, Pontificia Universidad Católica de Valparaíso, Avenida Universidad 330, Valparaíso 2373223, Chile; paz.caballero@pucv.cl (C.P.); jose.gallardo@pucv.cl (G.-M.J.); 2 Programa de Doctorado en Acuicultura, Pontificia Universidad Católica de Valparaíso, Universidad Católica del Norte and Universidad de Chile, Valparaíso 2373223, Chile; 3 Salmones Camanchaca S.A., Puerto Montt 5480000, Chilecsotov@camanchaca.cl (S.C.); 4 Escuela de Tecnología Médica, Pontificia Universidad Católica de Valparaíso, Avenida Universidad 330, Valparaíso 2373223, Chile; karin.martinez@pucv.cl

**Keywords:** Atlantic salmon, acute hypoxia, loss of equilibrium, hypoxia tolerance, hematological response, metabolic response, aquaculture

## Abstract

Hypoxia is a significant environmental and production-related stressor in Atlantic salmon aquaculture, yet the blood-based mechanisms underlying differential hypoxia tolerance remain poorly characterized. In this study, we examined hematological, biochemical, and transcriptional responses in Atlantic salmon parr classified as hypoxia-tolerant or hypoxia-susceptible following an acute progressive hypoxia challenge. Time to loss of equilibrium and dissolved oxygen concentration at loss of equilibrium were recorded. Blood samples were analyzed for hematological parameters, serum metabolites, and the expression of genes related to hypoxia signaling, glycolysis, oxidative stress, and immune regulation. Hypoxia-tolerant fish maintained equilibrium significantly longer than susceptible fish (Tolerant: 224 ± 18 min; Susceptible: 115 ± 24 min) and tolerated lower dissolved oxygen concentrations (Tolerant: 1.89 ± 0.09 mg L^−1^; Susceptible: 2.55 ± 0.34 mg L^−1^) before losing their ability to maintain equilibrium. Tolerance varied among families, while body weight was not significantly associated with time to loss of equilibrium. Acute hypoxia altered erythrocyte-related parameters, increased serum lactate (*p* < 0.0001), and reduced total protein (*p* = 0.0148) in both phenotypes. At the transcriptional level, tolerant fish exhibited higher expression of *hif1α* (*p* = 0.0012), *k6pp* (*p* = 0.0045) and *il15* (*p* = 0.0159), along with reduced *phd3* (*p* = 0.0317) expression, whereas susceptible fish showed increased *anx1* expression. Taken together, our results showed that hypoxia tolerance in Atlantic salmon is associated with a coordinated blood response involving metabolic compensation and hypoxia-responsive signaling, supporting the use of blood as a practical tool for identifying resilient fish.

## Simple Summary

One of the main environmental challenges affecting Atlantic salmon aquaculture is hypoxia, which can compromise fish welfare, growth, and survival. However, not all fish respond equally to hypoxia, and the biological mechanism that distinguishes more resilient individuals remains poorly understood. In this study, Atlantic salmon parr were exposed to an acute progressive hypoxia challenge and classified as hypoxia-tolerant or hypoxia-susceptible based on the time of loss of equilibrium. Tolerant fish maintained equilibrium for longer and endured lower oxygen concentrations before losing their ability to maintain equilibrium. Interestingly, this resilience to hypoxia is not related to body weight but differs between families. Blood analyses showed that acute hypoxia strongly altered oxygen transport and energy metabolism, with marked changes in hematological parameters. In addition, tolerant fish displayed a stronger molecular response involving hypoxia signaling, glycolysis, antioxidant defense, and immune-related regulation. Our findings indicate that blood can provide useful biomarkers for identifying Atlantic salmon with greater resistance to acute hypoxic stress. This information may support health monitoring and management strategies to improve fish welfare and sustainability in salmon aquaculture.

## 1. Introduction

Low dissolved oxygen is one of the most relevant environmental and production-related stressors affecting farmed fish [1,2,3,4,5,6]. In aquaculture, hypoxic events may arise from high temperature [7], algal blooms [8], and other ecological fluctuations that reduce oxygen availability [9,10]. Because oxygen supports aerobic metabolism, prolonged or severe reductions in dissolved oxygen can compromise the welfare of fish, limiting growth [10,11,12], disrupting physiological homeostasis [13], and increasing mortality [14]. An operational definition of hypoxia is 2 mg/L [15]. However, this value depends on variables such as species, and some studies identify values below this threshold as harmful concentrations for fish [16,17]. Interestingly, in salmonids, specifically rainbow trout (*Oncorhynchus mykiss*), this critical concentration value ranges from 1.22 to 2.80 mg/L [13]. In response to reduced oxygen, teleost fish activate physiological, hematological, biochemical, molecular, and behavioral responses aimed at maintaining oxygen supply and mitigating the negative effects of decreased ATP levels in tissues [13,16]. These responses include ventilatory and cardiovascular adjustments, modifications in oxygen transport capacity, alterations of acid–base balance, mobilization of energy substrates, activation of anaerobic pathways, and regulation of hypoxia-sensitive genes [13]. At the hematological level, changes in erythrocyte count, hemoglobin concentration, and hematocrit have been documented as compensatory mechanisms to improve oxygen transport capacity. In *Pelteobagrus fulvidraco* × *P. vachelli, Acipenser schrenckii* ♂ × *Acipenser baerii ♀* and *Pelteobagrus vachelli* acute hypoxia induces an initial increase in these parameters [3,18,19]; however, the response is not uniform. A study conducted on *Lophiosilurus alexandri* showed that physiological adaptation does not involve changes in hematological parameters [20]. Meanwhile, studies on *Danio rerio* [21] and *Phoxinus lagowskii* [22] report a decrease in erythrocyte count and hemoglobin levels under hypoxic stress conditions. On the other hand, at the biochemical level, hypoxia induces a reorganization of energy metabolism. An increase in plasma glucose content has been reported in *Salmo salar* [23]. Under hypoxic conditions, this may be associated with an increase in anaerobic metabolism, which is linked to higher plasma lactate concentrations, as has been observed in *Acipenser oxyrhynchus* [24] and *Clarias batrachus* [25] under hypoxic conditions. At the molecular level, these adaptive reactions are strongly associated with hypoxia-inducible factor 1 alpha (HIF-1α), which functions as a master regulator of the response to lowered oxygen availability [26]. Notably, *hif1α* is widely expressed across tissues of teleost fish [27], including *Salmo salar* [11,28].

Atlantic salmon (*Salmo salar*) is among the most important finfish species in global marine aquaculture, with worldwide production exceeding one million tonnes in 2023 [29]. In Chile, salmon farming represents the main activity in the aquaculture sector, with a total of 1,151,996 tonnes of salmon harvested in 2025, of which 797,592 tonnes correspond to Atlantic salmon [30]. Despite its high commercial value, this species is particularly vulnerable to reductions in dissolved oxygen availability [10]. Specifically, in Chile, salmon mortality associated with hypoxia was 4577 tonnes during 2025, equivalent to 45% of deaths caused by environmental factors and, interestingly, oxygen reduction events were reported in almost every month of the year [31]. Previous studies have documented marked intraspecific variation in hypoxia tolerance in *Salmo salar*, showing that different families within the same species can respond very differently to low-oxygen stress [32,33]. The mechanisms involved in the tolerance of *Salmo salar* are thought to be associated with metabolic adjustment [33] or an increase in myoglobin levels and cardiac alterations [32]. In addition to genetic background, body size may likewise influence hypoxia tolerance, as larger fish are generally considered more vulnerable to oxygen limitation because of their higher metabolic oxygen requirements and lower surface area-to-volume ratio, which may restrict oxygen uptake [14]. Such variability, which likely also occurs at the individual level, may reflect differences in oxygen transport efficiency, metabolic flexibility, stress responsiveness, and genetic background [13,32,34]. However, these studies have focused on a single tissue or examined only the metabolic effects of reduced oxygen levels [32,33], leaving the molecular and cellular mechanisms underlying differential tolerance unexplored. Furthermore, most research characterizing the response of *Salmo salar* to hypoxic stress has relied on genetic and transcriptional analyses of the gills, head kidney, spleen, and liver [5,35,36,37]. These approaches require sacrificing the animal, which limits the ability to fully characterize hypoxia effects under production conditions. This limitation is particularly significant given the increasing emphasis on developing more humane methodologies for animal research [38]. Moreover, the response to hypoxia is not exclusively metabolic, as immune and inflammatory pathways may also be modulated under low-oxygen conditions [5,36,39], showing the close interplay between energy allocation, cellular stress and physiological homeostasis.

In this context, blood serves as a particularly informative tissue for this purpose. Because blood circulates throughout the organism, it enables the collection of information from multiple biological systems [40]. Hematological cells provide direct insights into oxygen-carrying capacity and immune cell dynamics [41], while plasma metabolites can indicate acute energetic adjustments [40]. The primary cellular component in fish blood is the erythrocyte (98–99%), which is ellipsoidal and nucleated, in contrast to mammalian erythrocytes [42]. Notably, research in rainbow trout has demonstrated that erythrocytes express the *hif1α* gene [43]. This feature enables the acquisition of genetic information about hypoxia-related pathways, including glycolysis, redox state, and inflammation, as shown in studies of blood cell transcriptional profiles under hypoxic conditions in mammals [44]. The use of hematological and biochemical blood parameters has proven to be an effective and reproducible method for monitoring fish health, leading to their increasing adoption in aquaculture studies [41]. An integrated blood-based approach may therefore facilitate the identification of phenotypes that distinguish fish capable of maintaining function for longer periods under progressive hypoxia from those that succumb earlier.

In this study, we aimed to characterize blood-based differences between hypoxia-tolerant and hypoxia-susceptible Atlantic salmon subjected to an acute progressive hypoxia challenge. Our results showed that tolerant fish exhibit a more coordinated blood response characterized by prolonged functional resistance, greater energetic mobilization, and stronger induction of hypoxia-responsive pathways.

## 2. Materials and Methods

### 2.1. Experimental Fish and Husbandry

All experimental procedures were reviewed and approved by the Bioethics Committee of the Pontificia Universidad Católica de Valparaíso (PUCV), Valparaíso, Chile (approval code: BIOEPUCV-BA 738-2024; 17 April 2024).

Healthy Atlantic salmon (*Salmo salar*) parr from the Lochy strain (*n* = 223; mean body weight = 58.2 ± 19.1 g), provided by Salmones Camanchaca S.A. (Piscicultura Polcura, Comuna de Tucapel, Chile), were used in this study. This strain was selected because of its favorable production traits, including faster growth and a shorter production cycle [45]. The experimental population comprised 10 families. Fish were maintained in a 0.35 m^3^ open-flow tank under a 24:00 h (light:dark) regime at 5–7 °C, with a water flow rate of approximately 1 body length s^−1^. Scientific evidence shows that this photoperiod allows greater growth [46], as well as controlling maturation and smoltification processes [47]. Fish were fed with a commercial diet (protein: 47.2%, lipid: 23.4%, carbohydrates: 16.1%) to apparent or visual satiation. This strategy is used in production settings to maximize the growth of salmonids. Before the experimental procedures, all fish were acclimated to the experimental tanks for at least 14 days. Feeding was suspended for at least 24 h prior to the hypoxia challenge.

### 2.2. Acute Hypoxia Challenge and Phenotypic Classification

Two experimental conditions were evaluated: normoxia (~100% dissolved oxygen, DO) and hypoxia (<20% DO) and the experiment was conducted over two consecutive days: 6 August 2025 and 7 August 2025. In total, two tanks were assigned to the normoxia condition (N1: 6 August 2025, *n* = 36, mean body weight = 63.1 ± 19.4 g; N2: 7 August 2025, *n* = 37, mean body weight = 55.2 ± 18.2 g) and four to the hypoxia condition (H1: 6 August 2025, *n* = 36, mean body weight = 63.2 ± 16 g; H2: 7 August 2025, *n* = 37, mean body weight = 55.6 ± 18.7 g; H3: 7 August 2025, *n* = 37, mean body weight = 56.6 ± 19 g; H4: 7 August 2025, *n* = 37, mean body weight = 56.5 ± 19.2 g).

To minimize potential density-related stress in Atlantic salmon, culture biomass was maintained at approximately 17.5 kg m^−3^ in normoxia tanks and 18.0 kg m^−3^ in hypoxia tanks. This criterion was adopted because stocking density is a recognized stressor in Atlantic salmon, and cortisol production has been reported to increase at densities above 25 kg m^−3^ [40,48,49].

To assess hypoxia tolerance, fish were subjected to a progressive acute hypoxia challenge until loss of equilibrium (LOE). Time to loss of equilibrium (TLOE) was defined as the time elapsed from the onset of the trial, in minutes, until a fish lost its upright position and no longer responded to external stimuli [50]. Hypoxia was induced by injecting gaseous N_2_ every 5 min, resulting in a controlled decline in dissolved oxygen of approximately 0.8 mg L^−1^ during the first 15 min of the trial, followed by a phase of stability in which the oxygen concentration decreased by approximately 0.5 mg L^−1^ per interval, until reaching values of around 0.1 mg L^−1^ at the end of the trial (Supplementary Figure S1). Dissolved oxygen levels were progressively reduced from approximately 100% saturation (around 10 mg L^−1^) until the last fish lost equilibrium, which occurred between 1.57 and 1.98 mg L^−1^.

Because the assay was conducted in an open-flow freshwater facility (Polcura Pisciculture, Comuna de Tucapel, Chile), water inflow had to be stopped during the challenge. To maintain water movement within the tanks and prevent reduced fish mobility under stagnant conditions, an auxiliary pump provided constant internal circulation throughout the assay. This is an important consideration, as one of the behavioral changes observed in teleost fish involves entering a quiescent state, which allows them to conserve energy during hypoxic events [51]. For this reason, oxygen levels were measured at the bottom of the tank, since this was the area where the fish were predominantly located during the trial.

**Figure 1:**
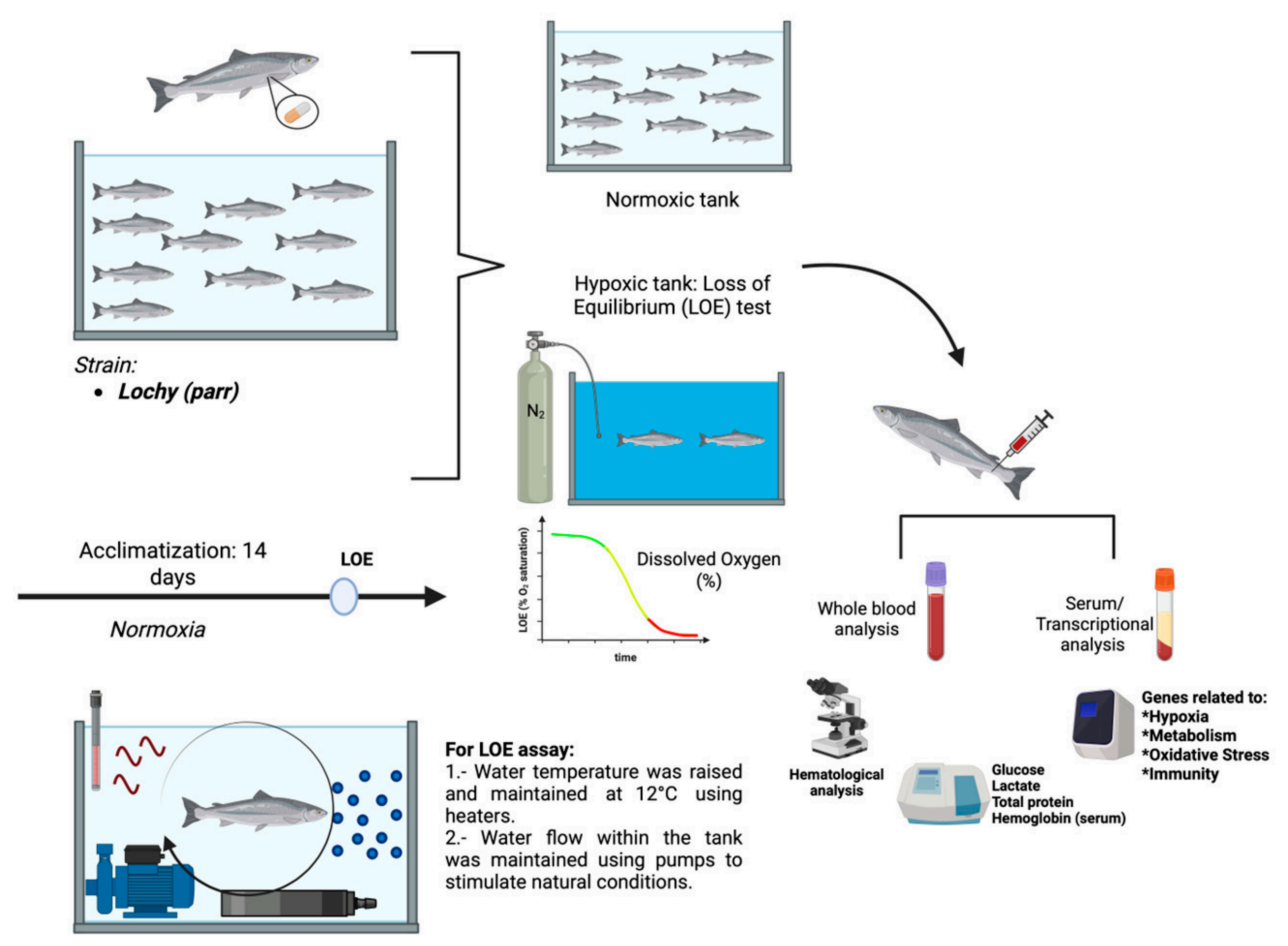
Experimental process for Atlantic salmon parr (*Salmo salar*) under acute hypoxia. The colors represent the different zones and the potential physiological effects that these oxygen levels may have on the fish: green (no adverse effects), yellow (adverse effects may occur), and red (the fish experiences adverse effects). In addition, the blue circles in the tank represent the release of nitrogen, whereas the red wavy lines represent the heat emitted by the heater. Created with Biorender (https://www.biorender.com).

Since temperature has also been reported to influence hypoxia tolerance [52,53], the trial was conducted at 12 °C. This temperature was selected because Atlantic salmon can grow within a range of approximately 8 to 14 °C [54,55]. As water temperature at the pisciculture ranged from approximately 5 °C at night to 7 °C during the day, water was gradually warmed using aquarium heaters until the target temperature was reached (Figure 1). Temperature and dissolved oxygen were monitored every 5 min using a Polaric C dissolved oxygen meter (OxyGuard^®^, Farum, Denmark), allowing us to obtain this information prior to the injection of N_2_. The temperatures recorded in the tanks during the experiment were as follows: 12.5 ± 0.1 °C (N1), 12.1 ± 0 °C (N2), 12.5 ± 0.05 °C (H1), 12.1 ± 0 °C (H2), 12.0 ± 0 °C (H3), and 12.0 ± 0 °C (H4).

Each fish carried an individual electronic tag, enabling longitudinal identification and full traceability throughout the trial. At the endpoint, TLOE and dissolved oxygen concentration were recorded for each fish. Fish that maintained equilibrium until the final stage of the assay and lost it at dissolved oxygen concentrations below 2 mg L^−1^ were classified as hypoxia-tolerant, whereas those that lost equilibrium earlier, at dissolved oxygen concentrations above 2 mg L^−1^, were classified as hypoxia-susceptible. The use of 2 mg L^−1^ dissolved oxygen as the threshold for classification was based on established definitions of hypoxia in fish. Additionally, Barnes et al. [33] reported a value of 1.98 mg/L for *Salmo salar* with an average weight of 47.2 g at a temperature of 15.6 ± 0.5 °C. Once the fish lost their equilibrium, they were classified as either susceptible or tolerant according to the oxygen concentration, and tissue samples were collected immediately.

### 2.3. Family and Body Size Analysis

To determine whether hypoxia tolerance was associated with genetic background, TLOE was compared among ten Atlantic salmon families derived from 15 broodstock fish. The experimental design included ten dams and five sires, with each sire crossed with two dams, resulting in ten families arranged as five pairs of paternal half-sibling families (Table 1). Thus, families within each pair shared the same sire but had different dams, whereas families from different pairs were unrelated. Family representation was balanced at the start of the experiment by randomly distributing fish from the different families across the experimental groups. Body length was measured to the nearest millimeter from the tip of the snout to the end of the middle caudal-fin rays, and body weight was determined using a digital balance with an accuracy of 0.01 g. This value was used to calculate the k index.

### 2.4. Anesthesia and Blood Sampling

Fish classified as hypoxia-susceptible or hypoxia-tolerant were euthanized before blood collection by overdose with benzocaine (200 mg L^−1^; Sigma-Aldrich, St. Louis, MO, USA), following animal welfare guidelines. Stress and anesthesia are known to alter physiological blood parameters [42]. Therefore, blood samples were collected exclusively from fish that were immediately classified as tolerant or susceptible, within a maximum of 3 min [56]. Fish that could have remained anesthetized for extended periods were excluded from sampling. Due to the methodology and classification system, large numbers of fish occasionally lost balance and accumulated, rendering them unsuitable for blood sampling. Consequently, only a limited number of fish were included in the blood analysis (10 fish in total). Furthermore, because of the small body size and limited blood volume of each fish, separate individuals were used for hematological and serum biochemical or transcriptional analyses. As a result, these datasets were treated as parallel subsets rather than repeated measurements from the same individuals. Finally, given the distance between the trial site and the sample processing facility (over 600 km) and the restricted timeframe for metabolite analysis specified in the detection kit manual (1–3 days), blood was collected exclusively on the second day of the trial.

Blood was collected from the caudal vein using sterile syringes and immediately placed on ice. For hematological and whole-blood analyses, samples were collected to a final volume of 500 µL and transferred to 500 µL non-vacuum K2-EDTA tubes (BD Microtainer^®^, Franklin Lakes, NJ, USA). For serum analyses, blood samples were collected to 500–800 µL into clot-activator tubes with separator gel (Vacsure, CML Biotech Ltd., Angamaly, Kerala, India). All samples were transported to the laboratory (Valparaíso region, Chile) under refrigerated conditions. For serum preparation, blood was centrifuged at 1500× *g* for 5 min, and the supernatant was transferred to labeled tubes. All samples were processed within 24–48 h after collection. For transcriptional analysis, 200 µL of blood from fish allocated to serum determinations was preserved in 500 µL of RNAlater (Life Technologies, Carlsbad, CA, USA), kept at 4 °C for 24 h, and then stored at −80 °C until processing.

### 2.5. Hematological Analysis

The hematological parameters analyzed in this study included hematocrit (Hct), red blood cell (RBC), hemoglobin concentration (Hgb), mean corpuscular volume (MCV), mean corpuscular hemoglobin concentration (MCHC), white blood cell count (WBC), mid-size cells (MID), granulocytes (GRA) and lymphocyte abundance (LYM). Hemoglobin concentration was determined by the cyanmethemoglobin method, and MCV using a HumaCount 80TS (HUMAN, Wiesbaden, Hesse, Germany). Hematocrit was measured by centrifuging blood-filled microcapillary tubes at 12,000 rpm for 15 min at room temperature. RBC was estimated indirectly using the following equation: RBC = (Hct × 10)/(MCV × 1000), with results expressed as cells × 10^7^ L^−1^. This approach was necessary because fish erythrocytes are nucleated cells and therefore cannot be accurately identified by automated hematology analyzers. White blood cell counts were determined according to the method of Walberg [57], with modifications. Briefly, leukocytes were counted by microscopic observation at 100× magnification in ten consecutive fields, and the average value obtained was multiplied by a factor of 1000 to express the results as absolute cells per microliter. For morphological evaluation, blood smears were prepared and stained using the May–Grünwald–Giemsa method. Differential leukocyte counts were performed by microscopic examination of 100 consecutive leukocytes per sample, classifying each cell as a lymphocyte, granulocyte, or monocyte [58] Microscopic evaluation followed the principles of the reference manual leukocyte differential count described in the CLSI H20 guideline and standard blood smear examination protocols [59]. The resulting leukocyte proportions were expressed as relative percentages. The 100-cell differential is the conventional approach for manual blood smear evaluation and is consistent with veterinary hematology references and studies addressing the analytical uncertainty of manual leukocyte differentials [58,60]. Secondary hematimetric indices were calculated according to Wintrobe’s formulas: MCHC was obtained from the relationship between hemoglobin concentration and absolute hematocrit.

### 2.6. Biochemical Blood Parameters

Commercial kits were used to measure the concentrations of glucose (GOD-POD liquid, Spinreact, Sant Esteve de Bas, Girona, Spain), lactate (LO-POD Enzymatic colorimetric, Spinreact, Sant Esteve de Bas, Girona, Spain), total protein (Pierce™️ BCA Protein Assay Kits, ThermoFisher, Waltham, MA, USA) and hemoglobin (Hemoglobin Assay Kit, Sigma-Aldrich, St. Louis, MO, USA) in the serum samples, following the manufacturers’ instructions.

### 2.7. Total RNA Extraction, cDNA Synthesis and Gene Expression Analysis

For primer design, the sequences of the genes of interest were obtained from the NCBI/nucleotide database (https://www.ncbi.nlm.nih.gov). Primer pairs were designed and analyzed using the Primer-BLAST tool (https://www.ncbi.nlm.nih.gov/tools/primer-blast/ accessed on 18 May 2025) and Oligo Analyzer (https://www.idtdna.com/pages/tools/oligoanalyzer accessed on 18 May 2025). Subsequently, the specificity of the primers was evaluated using the nucleotide BLAST tool (https://blast.ncbi.nlm.nih.gov/Blast.cgi accessed on 18 May 2025). Primers were synthesized by Integrated DNA Technologies (IDT, Coralville, IA, USA).

Total RNA was isolated using TRIzol^®^ reagent (Invitrogen, Carlsbad, CA, USA). Briefly, blood cells stored in RNAlater were centrifuged at 7500× *g* for 10 min at 4 °C, and the resulting pellet was divided into two portions. One portion was resuspended in 1000 µL of TRIzol^®^, and cells were lysed using a tuberculin syringe followed by vortex homogenization. RNA extraction was then completed according to the manufacturer’s instructions. RNA concentration was determined using an ND-1000 spectrophotometer NanoDrop® LITE spectrophotometer (Thermo Scientific, Waltham, MA, USA), with the following results obtained for each experimental group: Normoxia (58.7 ± 18.7 ng/mL; Abs260/280: 2 ± 0.2); Susceptible (55.2 ± 8.5 ng/mL; Abs260/280: ~2 ± 0.1); and Tolerant (59.4 ± 20.9 ng/mL; Abs260/280: 2 ± 0.2). RNA integrity was evaluated by agarose gel electrophoresis. Extracted RNA was stored at −80 °C until further analysis.

For cDNA synthesis, approximately 300 ng of total RNA was reverse transcribed using the RevertAid First Strand cDNA Synthesis Kit (Thermo Scientific, Waltham, MA, USA) according to the manufacturer’s protocol. Quantitative PCR was performed with KAPA SYBR^®^ FAST (KAPABIOSYSTEMS, Wilmington, MA, USA) in an AriaMx qPCR System (Agilent, Santa Clara, CA, USA). The thermal profile included an initial step at 95 °C for 3 min, followed by 40 cycles of 95 °C for 5 s, 60 °C for 15 s and 72 °C for 15 s, with fluorescence measured at the end of each annealing/extension step. *elf1α* and *hprt* [61] were used as reference genes, and the relative expression of *hif1α*, *phd3*, *k6pp*, *ldhb*, *il15*, and *anx1* (Table 2) was determined using the 2^−ΔΔCt^ method using the mean of both reference genes [62].

### 2.8. Statistical Analysis

Statistical analyses and graphical outputs were performed using GraphPad Prism 10, whereas normalization by experimental day and tank, together with the graphical representation of normalized data (family vs. TLOE), was conducted in R (version 4.5.3). Descriptive data are presented as means ± standard deviation (SD). Potential outliers were screened using Grubbs’ test and excluded only when statistically supported. Prior to inferential analyses, data distribution was assessed using the Shapiro–Wilk test, and homogeneity of variance was evaluated accordingly. Transcriptional data, together with comparisons of TLOE and dissolved oxygen concentration between hypoxia-tolerant and hypoxia-susceptible fish, were analyzed using an unpaired two-tailed t-test or Mann–Whitney test, as appropriate. Biochemical and whole-blood variables were analyzed using one-way ANOVA, and these analyses were followed by Tukey’s multiple comparison post hoc test. Effect size was reported as eta squared for the t-test and R squared for the ANOVA analysis. For the non-parametric Mann–Whitney test, the value of r was manually calculated using the formula r = Z/√N [63], based on the Mann–Whitney U value provided by Graphpad Prism 10.

To evaluate the effect of family on LOE time, values were normalized according to the trial day and the tank to reduce potential bias associated with experimental time and tank-specific effects. For this purpose, each fish was assigned to a stratum based on the following combination: trial date x tank. Subsequently, for each combination, the mean and standard deviation of TLOE in minutes were calculated, allowing for the characterization of the behavior of each tank/day. Then, a normalized value was calculated for each fish using the following formula:

$$z=\frac{TLOEi-Xstratum}{SDstratum}$$

where

- *TLOEi*: TLOE of fish *i*
- *“X” stratum*: mean of the stratum (date × tank)
- *“SD” stratum*: standard deviation of the stratum

As the sample sizes among families were unequal, a non-parametric test (Kruskal–Wallis) and post hoc multiple comparisons were applied (Holm). Z-score normalization is a statistical technique that facilitates the comparison of results across different datasets or variables, and it has demonstrated superior performance compared to other methodologies [64].

To evaluate the effect of weight on LOE time, we use simple linear regression, with body weight (g) as the independent variable and normalized TLOE (TLOE Z-score) as the dependent variable. Statistical significance was established at *p* < 0.05.

## 3. Results

### 3.1. Tolerant Fish Maintained Function Longer Under Hypoxia

**Figure 2:**
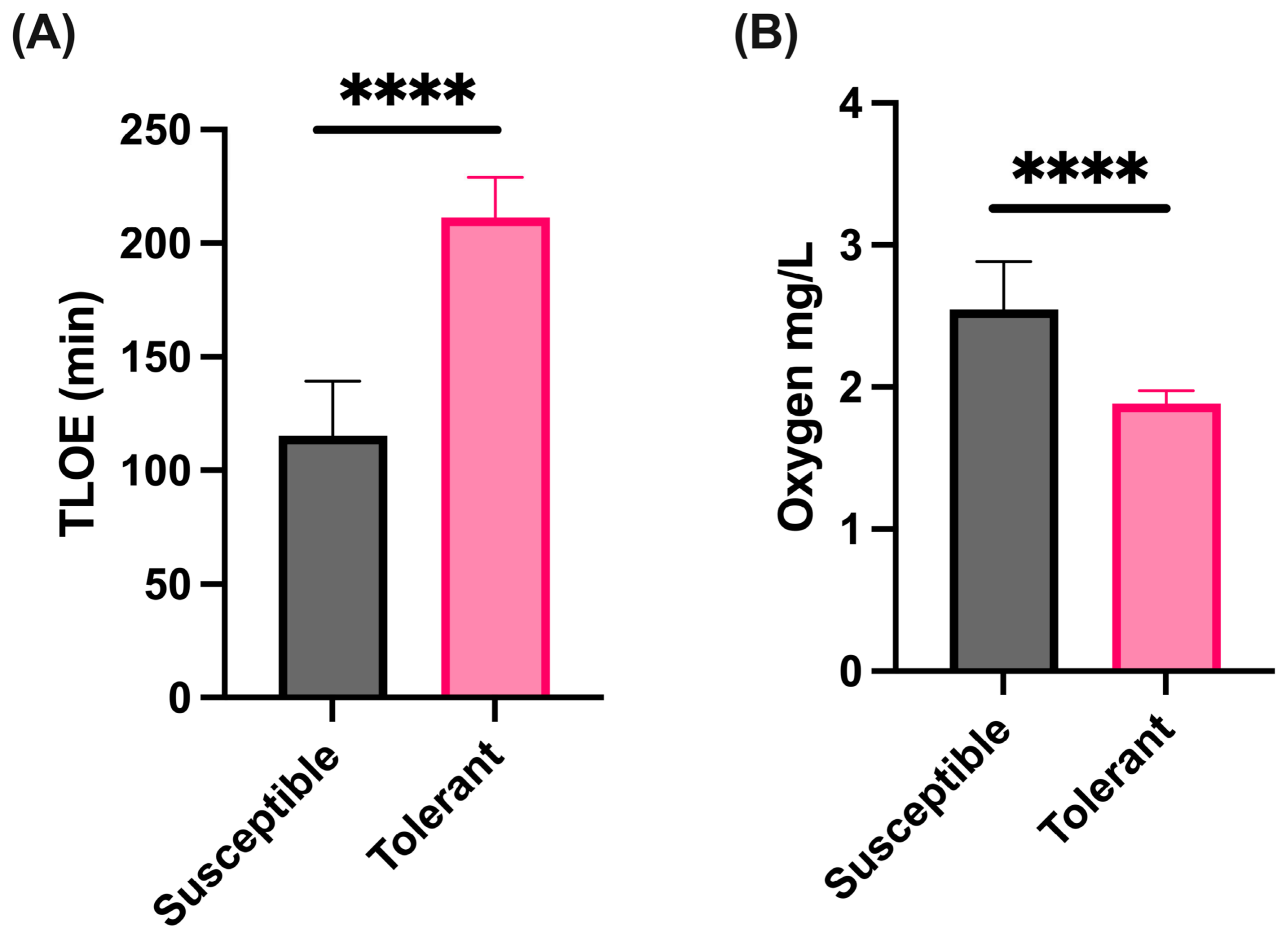
Acute hypoxia challenge shows two distinct phenotypes in Atlantic salmon parr (*Salmo salar*). TLOE (**A**) and oxygen concentration (**B**) differences between susceptible and tolerant classified fish. Data are presented as mean ± SD (*n* = 10 fish per group). Asterisks indicate significant differences relative to the control group (**** *p* < 0.001).

The acute hypoxia challenge clearly separated Atlantic salmon into distinct phenotypes according to their resistance to oxygen depletion. Hypoxia-tolerant fish maintained equilibrium significantly longer than hypoxia-susceptible fish (*p* < 0.0001, effect size = 0.8518), with a TLOE of 224 ± 18 min compared with 115 ± 24 min in susceptible individuals (Figure 2A). Moreover, tolerant fish reached significantly lower dissolved oxygen concentrations before loss of equilibrium (*p* < 0.0001, effect size = 0.85), averaging 1.89 ± 0.088 mg L^−1^ compared with 2.55 ± 0.338 mg L^−1^ in susceptible fish (Figure 2B). Together, these results suggest that tolerant fish preserved functional capacity under more severe oxygen limitation.

### 3.2. Hypoxia Tolerance Varied Among Families, but It Is Not Related to Body Weight

**Figure 3:**
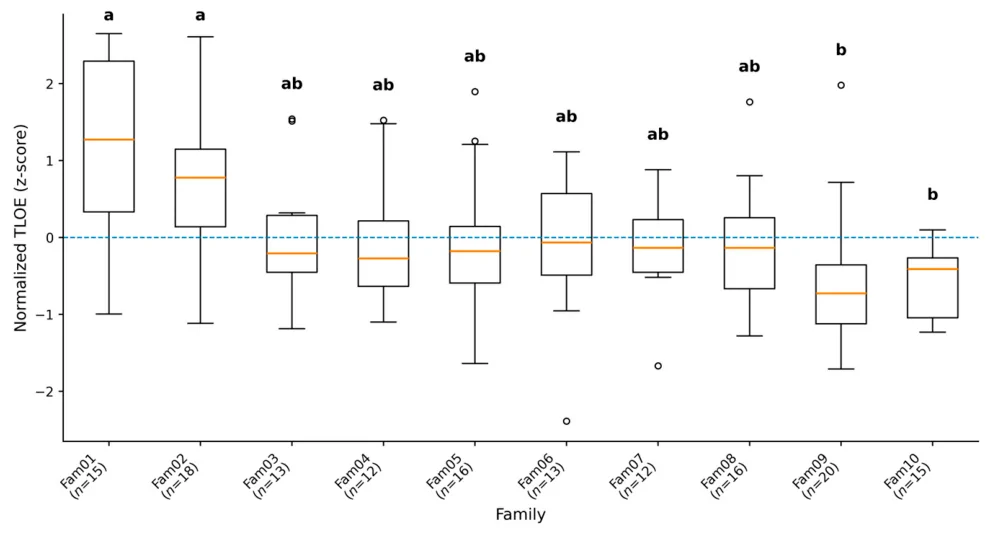
Family-specific variation in normalized time to loss of equilibrium (TLOE) in the Lochy strain of Atlantic salmon (*Salmo salar*). Boxplots display normalized TLOE values across families. Sample sizes for each family are shown on the *x*-axis. Different letters indicate statistically significant differences among groups after post hoc multiple comparison testing; groups sharing at least one letter are not significantly different. The orange line represents the mean value, while the circles correspond to individual members of the family whose values deviate from the overall trend. Finally, the blue dashed line was used solely to indicate the zero value on the graph.

**Figure 4:**
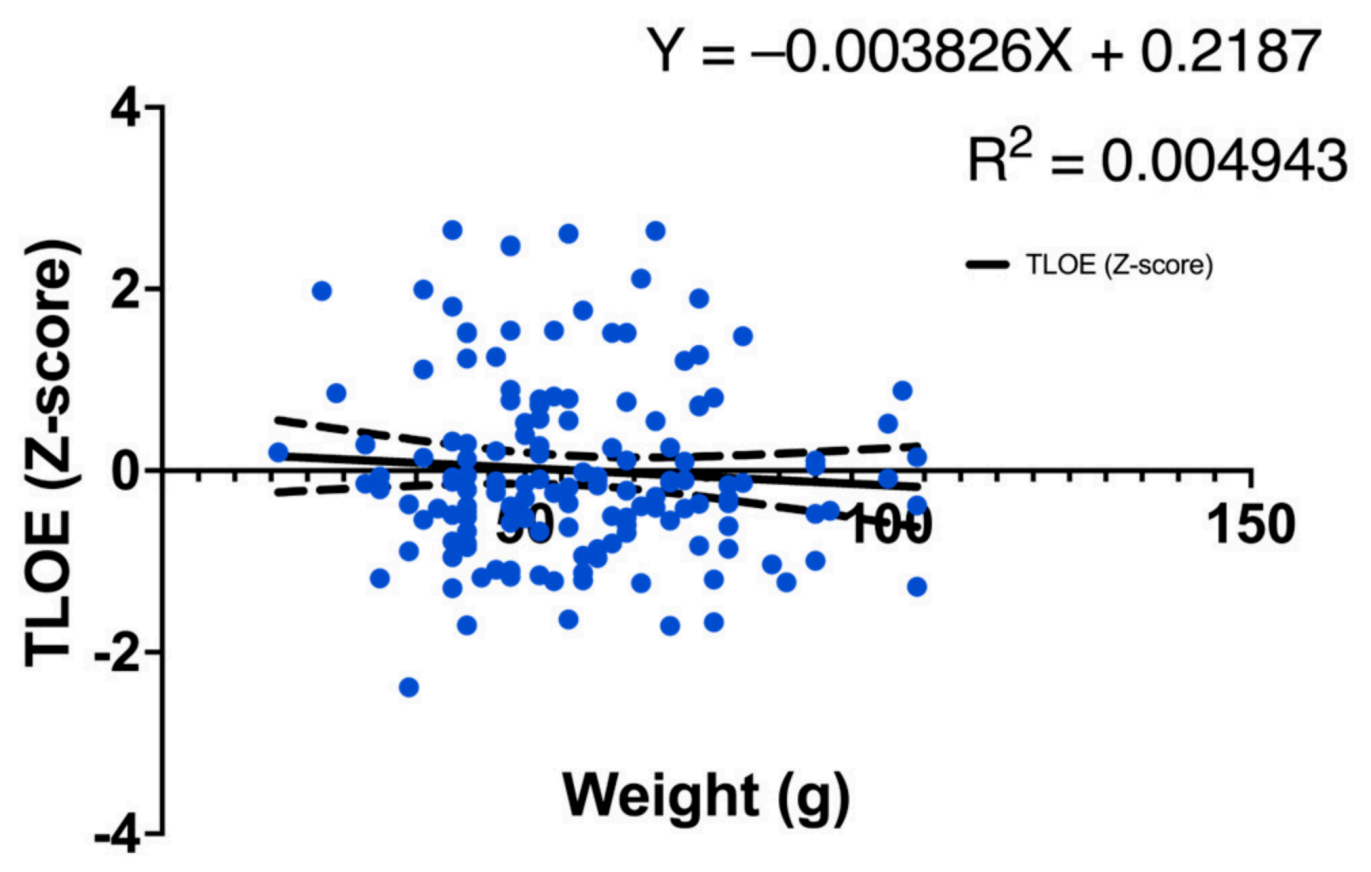
Relationship between body weight and TLOE in the Lochy strain of Atlantic salmon (*Salmo salar*). Each point represents an individual fish (*n* = 150). The solid line represents the simple linear regression, and the dot lines indicates the 95% confidence interval.

After normalization of TLOE values (Table 3), significant family-related differences were detected (*p* < 0.001). Families Fam01 and Fam02 showed significantly higher normalized TLOE values than families Fam09 and Fam10 (Figure 3). In contrast, simple linear regression showed no significant association between body weight and TLOE (R^2^ = 0.0049, *p* = 0.393) (Figure 4). Taken together, these results suggest that hypoxia tolerance is more strongly associated with family background/individual differences than with body mass. 

### 3.3. Hematological Changes Indicate Marked Erythrocyte Disturbance Under Acute Hypoxia

**Figure 5:**
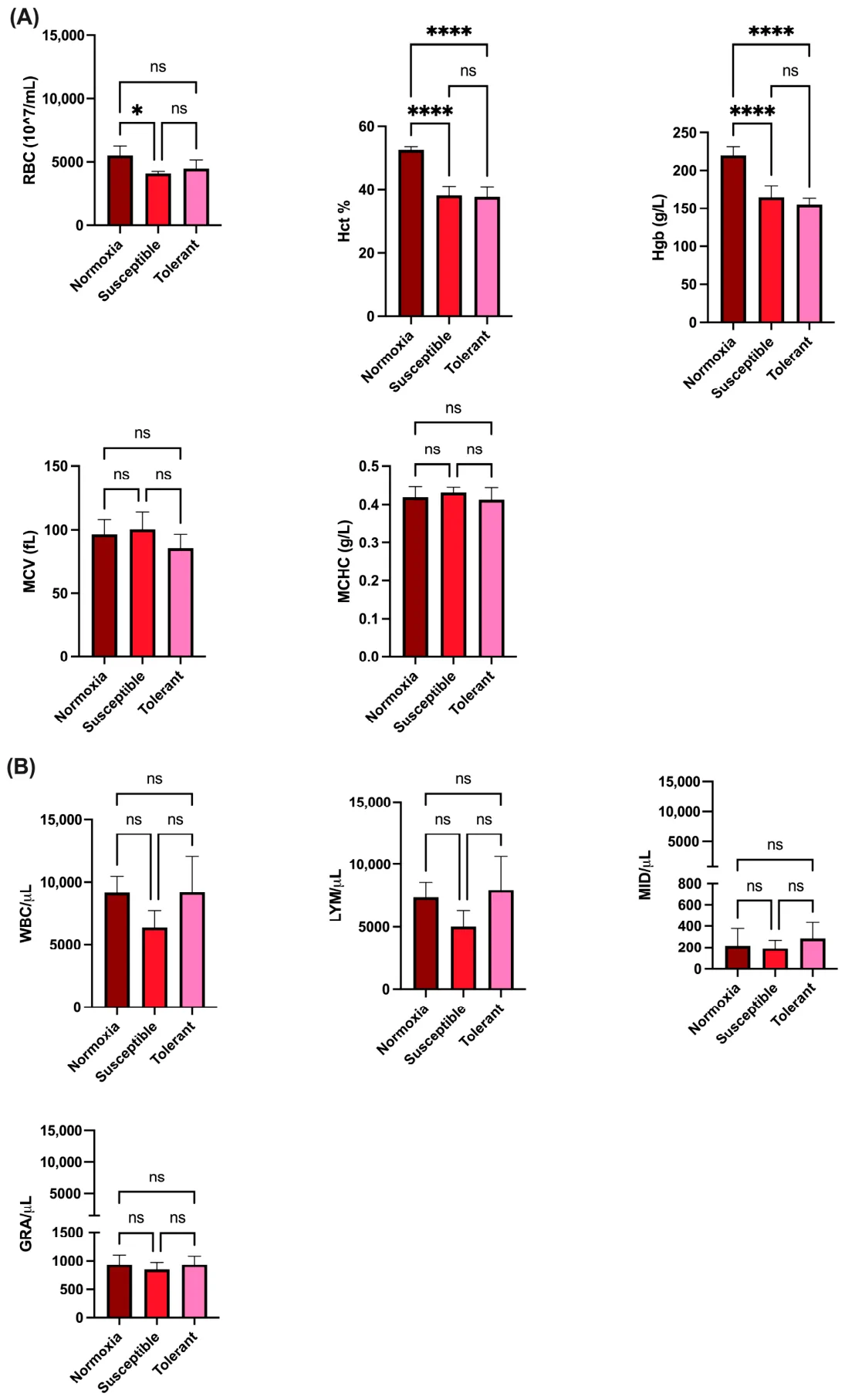
Effects of acute hypoxic stress on (**A**) hematological parameters and (**B**) leukocyte-related parameters in Atlantic salmon parr (*Salmo salar*). Exposure to low DO altered parameters associated with oxygen transport capacity. Data are presented as mean ± SD (*n* = 4–5 fish per group). Asterisks indicate significant differences relative to the control group (* *p* < 0.05; **** *p* < 0.0001); ns is non-significant.

Acute hypoxia significantly affected several hematological variables. RBC abundance differed among groups (*p* = 0.0234, effect size = 0.5282), with the highest mean in normoxic fish (5505 ± 793 × 10^7^ mL^−1^), the lowest in susceptible fish (4109 ± 158 × 10^7^ mL^−1^), and an intermediate value in tolerant fish (4482 ± 673 × 10^7^ mL^−1^). Hct was strongly reduced in both hypoxia-classified groups relative to normoxia (*p* < 0.0001, effect size = 0.8922), declining from 53 ± 1% in normoxia to 38 ± 3% in both susceptible and tolerant fish. Hgb showed a similar pattern (*p* < 0.0001, effect size = 0.8679), decreasing from 220 ± 12 g L^−1^ in normoxia to 165 ± 15 g L^−1^ in susceptible fish and 155 ± 8 g L^−1^ in tolerant fish. However, MCV (*p* = 0.1765, effect size = 0.2705) and MCHC (*p* = 0.5217, effect size = 0.1116) remained statistically unchanged across groups (Figure 5A).

Weaker responses were observed in leukocyte-related variables. Total leukocyte count tended to differ among groups, but did not reach statistical significance (*p* = 0.0844, effect size = 0.3620), with susceptible fish showing the lowest mean (6360 ± 1376 µL^−1^) compared with normoxia and tolerant fish. A similar tendency was observed for LYM (*p* = 0.0716, effect size = 0.3809), with the lowest values again in susceptible fish (5003 ± 1319 µL^−1^). In contrast, MID (*p* = 0.5292, effect size = 0.1093) and GRA (*p* = 0.6170, effect size = 0.1018) counts did not change significantly between groups (Figure 5B). Together, these data suggest that acute hypoxia was primarily associated with marked changes in erythrocyte-related parameters.

### 3.4. Serum Physiological Profiling Revealed Pronounced Metabolic Disturbance Under Hypoxia

**Figure 6:**
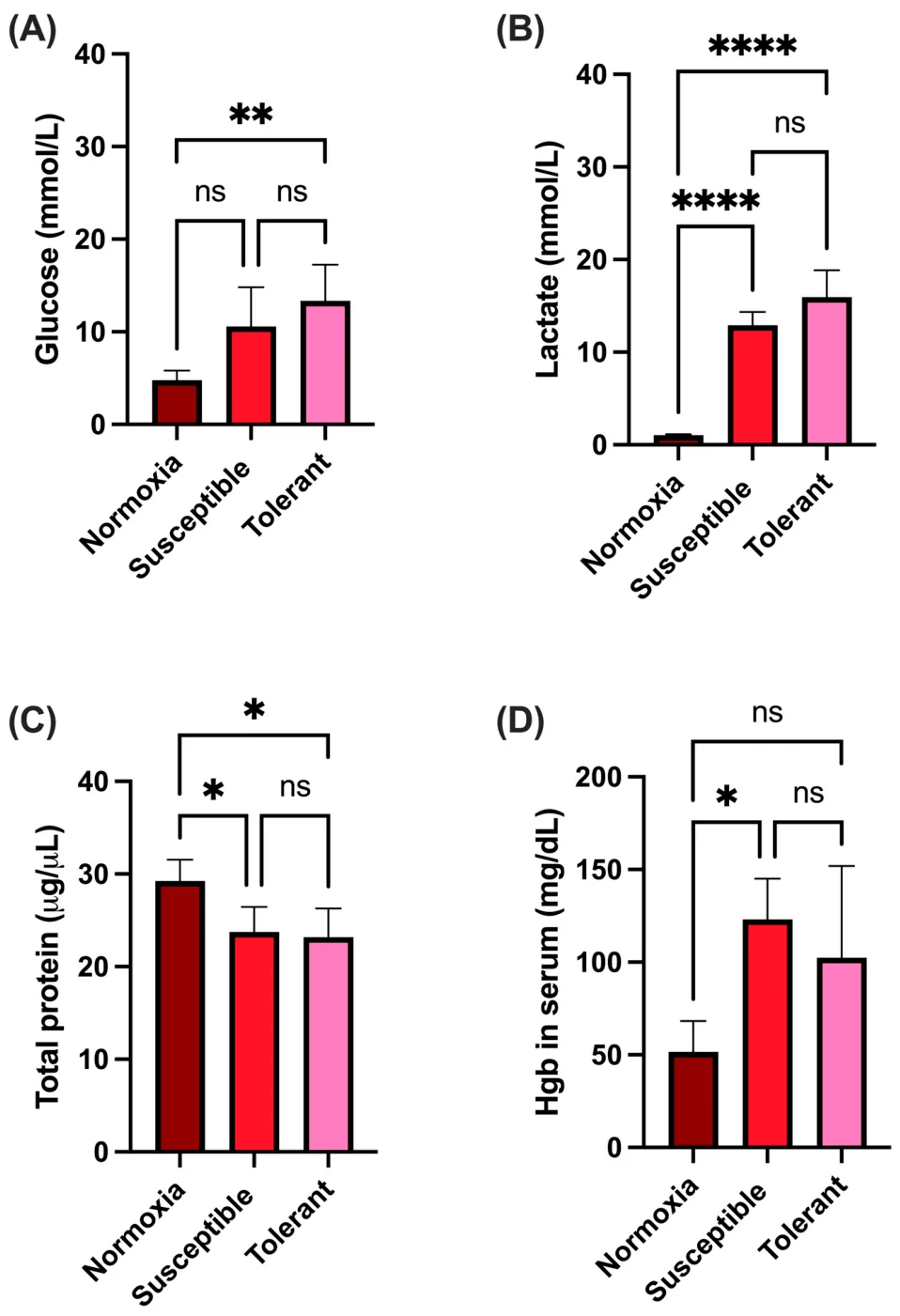
Effects of acute hypoxic stress on serum glucose (**A**), lactate (**B**), total protein (**C**), and serum hemoglobin concentrations (**D**) in Atlantic salmon parr (*Salmo salar)* classified as hypoxia-tolerant or hypoxia-susceptible. Data are presented as mean ± SD (*n* = 4–5 fish per group). Asterisks indicate significant differences from the control group (* *p* < 0.05; ** *p* < 0.01; **** *p* < 0.0001); ns is non-significant.

Serum biochemical variables were significantly altered by acute hypoxia, as shown in Figure 6. Glucose differed among groups (*p* = 0.0120, effect size = 0.5526), increasing from 4.769 ± 1.061 mmol L^−1^ in normoxia to 10.580 ± 4.221 mmol L^−1^ in susceptible fish and reaching the highest mean in tolerant fish (13.356 ± 3.901 mmol L^−1^). Lactate showed the strongest response among the biochemical markers analyzed (*p* < 0.0001, effect size = 0.9291), rising sharply from 1.041 ± 0.093 mmol L^−1^ in normoxia to 12.924 ± 1.424 mmol L^−1^ in susceptible fish and 15.935 ± 2.926 mmol L^−1^ in tolerant fish. Total protein was significantly reduced under hypoxia (*p* = 0.0148, effect size = 0.5352), decreasing from 29.243 ± 2.319 µg µL^−1^ in normoxia to 23.748 ± 2.687 µg µL^−1^ in susceptible fish and 23.189 ± 3.099 µg µL^−1^ in tolerant fish.

Hemoglobin measured in serum also differed between groups (*p* = 0.0454, effect size = 0.4971), with markedly higher values in hypoxia-exposed fish than in normoxic controls (Figure 6D). In summary, these data suggest that acute hypoxia-induced substantial metabolic disruption in both phenotypic groups, marked by higher lactate levels and reduced total protein levels.

### 3.5. Tolerant Fish Displayed a Distinct Hypoxia-Responsive and Immune-Related Transcriptional Signature

**Figure 7:**
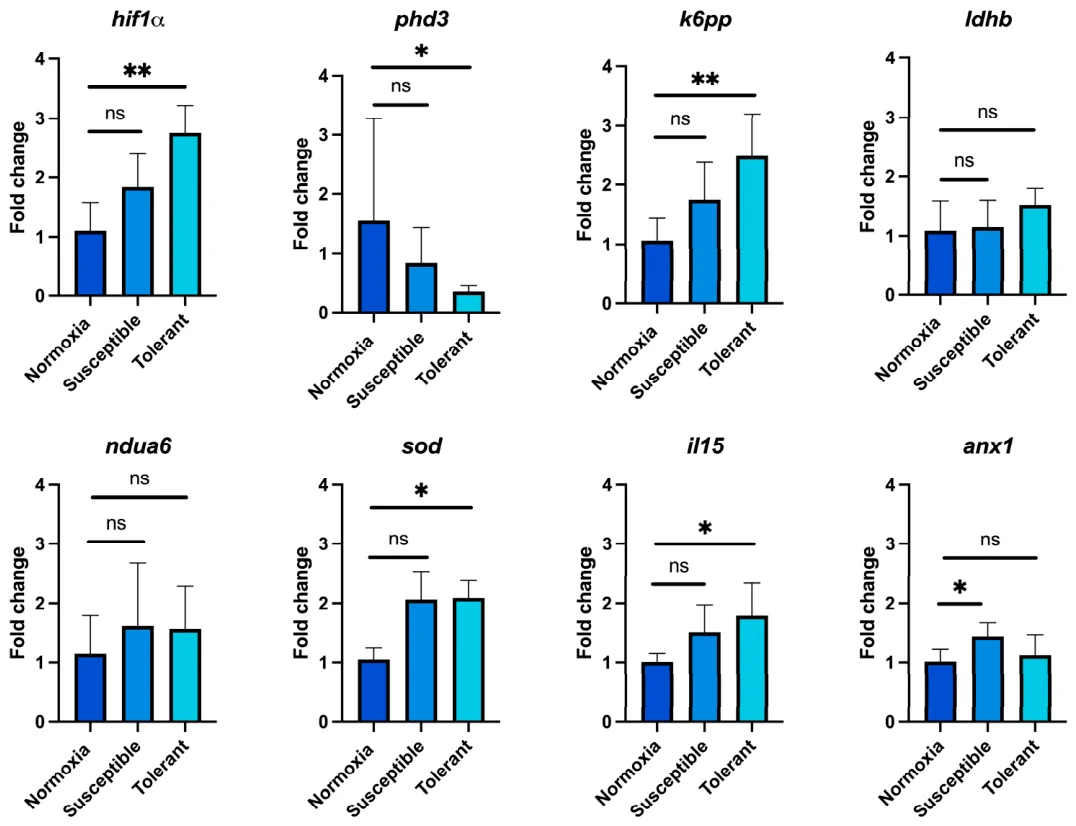
Acute hypoxia-induced expression of genes related to the HIF pathway, glycolysis, oxidative stress, and immune response in Atlantic salmon parr (*Salmo salar*). Fish classified as hypoxia-tolerant showed a distinct expression profile with a more pronounced metabolic response. Results are presented as mean ± SD (*n* = 5 fish per group) (* *p* < 0.05; ** *p* < 0.01 vs. control group); ns is non-significant.. *hif1α*: hypoxia-inducible factor 1 alpha; *phd3*: Prolyl hydroxylase domain 3; *k6pp*: ATP-dependent 6-phosphofructokinase; *ldhb*: Lactate dehydrogenase b; *ndua6*: NADH dehydrogenase ubiquinone 1 alpha subcomplex subunit 6; *sod*: Superoxide dismutase; *il15*: Interleukin 15; *anx1*: Annexin 1.

Gene expression analysis of blood cells revealed changes in several hypoxia- and immune-related transcripts as it shown in Figure 7. *hif1α* expression differed significantly between normoxia and tolerant fish (*p* = 0.0012, effect size = 0.7962), increasing from 1.105 ± 0.470 in normoxia to 2.753 ± 0.469 in tolerant fish. A similar pattern was observed for *k6pp*, which was significantly upregulated in tolerant fish compared with normoxia (*p* = 0.0045, effect size = 0.6566), increasing from 1.063 ± 0.377 to 2.482 ± 0.719. Interestingly, susceptible fish showed an intermediate mean value. In contrast, *phd3* expression was significantly reduced in tolerant fish relative to normoxia (*p* = 0.0317, effect size = 0.73).

Among the remaining metabolic genes, *ldhb* (Susceptible *p* = 0.8417, effect size = 0.005; Tolerant *p* = 0.1267, effect size = 0.2664) and *ndua6* (Susceptible p = 0.4166, effect size = 0.084; Tolerant *p* = 0.3526, effect size = 0.1085) showed higher expression in hypoxia-exposed fish than in normoxic controls, but these differences were not statistically significant. By contrast, *sod* expression was significantly elevated in tolerant fish (*p* = 0.0265, effect size = 0.5284), rising from 1.052 ± 0.396 to 2.092 ± 0.647. However, expression did not differ significantly from that in susceptible fish, likely due to the high variability observed in the susceptible group. Immune-related markers also showed differential regulation. *il15* expression was significantly higher in tolerant fish than in normoxic controls (*p* = 0.0159, effect size = 0.82). In contrast, *anx1* expression was significantly higher in susceptible fish than in normoxic controls (*p* = 0.0218, effect size = 0.5521). Overall, these data suggest that the tolerant phenotype was associated with activation of the HIF pathway.

## 4. Discussion

Hypoxia is a major environmental and production stressor in aquaculture, as even small fluctuations in dissolved oxygen can impair aerobic metabolism, reducing growth, compromising fish health and increasing mortality risk in farmed species [10,11,12,14]. However, fish show varying degrees of tolerance to this stressor, which can differ between species, families and individuals. This variation probably arises from interactions among multiple factors, including temperature, salinity, oxygen transport capacity, metabolic demand and biochemical plasticity [13,65]. The present study shows that Atlantic salmon (*Salmo salar*) display individual and family-level variation in acute hypoxia tolerance, as measured by time to loss of equilibrium (TLOE), and that this phenotypic variation is associated with distinct hematological, biochemical and transcriptional profiles.

Loss of equilibrium (LOE) has become a widely used, robust endpoint for assessing hypoxia tolerance in fish [66]. Importantly, longer TLOE has been associated with improved subsequent survival, supporting its ecological and biological relevance as an indicator of extreme hypoxia resistance [67]. In the present study, we observed approximately a two-fold difference in TLOE between hypoxia-tolerant and hypoxia-susceptible fish, consistent with previous reports of substantial inter-individual variation in hypoxia tolerance within salmonid populations [17,32]. However, a key limitation of the LOE endpoint is that it primarily captures tolerance at the severe end of the oxygen spectrum, rather than earlier sublethal impairment. This distinction is important because hypoxia is commonly defined as a reduction in dissolved oxygen to 2 mg L^−1^, whereas negative effects on growth and feed intake have been reported at higher oxygen levels, depending on the species and experimental context [17]. This may be of particular interest to Chile, where an oxygen minimum zone (OMZ) has been identified in the Los Lagos Region, an area characterized by intensive salmon farming [68]. Its relevance is further heightened this year by the occurrence of the El Niño–Southern Oscillation (ENSO), whose warm phase is a climate event associated with the Humboldt Current System (HCS) and contributes to increased water temperatures [69]. This rise in temperature is particularly concerning because dissolved oxygen availability is temperature-dependent [13], with warmer waters retaining less oxygen, thereby potentially increasing the risk of hypoxia under farming conditions. Notably, increasing temperatures have been observed not only in marine environments but also in Chilean lakes, a pattern that may be associated with global warming and rising air temperatures [70]. These temperature changes in both freshwater and seawater environments are relevant throughout the salmon production cycle, as scientific evidence indicates that seawater performance depends on previous freshwater rearing conditions, including whether fish are raised in open-flow or recirculating systems [71]. In our study, the use of parr-stage fish, which were fasted for at least 24 h prior to the assay, may partly explain why even fish classified as hypoxia-susceptible were able to tolerate relatively low DO levels before losing equilibrium. This interpretation is also consistent with wider comparative evidence indicating that freshwater fishes are often more hypoxia-tolerant than marine fishes, likely because they have evolved in more variable oxygen and temperature environments [72]. In addition, micro-scale position within the tank may have contributed to variation in response, since oxygen availability can differ locally as fish exploit better-oxygenated zones or surface-associated water layers, particularly during progressive hypoxia [66]. Therefore, the operational classification used here—susceptible fish losing equilibrium above 2 mg L^−1^ and tolerant fish doing so below 2 mg L^−1^—appears justified as a relative phenotypic framework within this experimental system, even if biologically relevant hypoxic stress probably began earlier along the oxygen-decline trajectory.

Another important finding of this study was that hypoxia tolerance varied among families but was not significantly associated with body mass. This result aligns with previous work by Anttila et al. [32], who showed that family-level variation in combined temperature–hypoxia tolerance in Atlantic salmon was linked to differences in relative ventricle mass and myoglobin concentration. Similarly, Scott et al. [73] found significant genetic variation in hypoxia tolerance among rainbow trout strains, further supporting the view that hypoxia tolerance has a genetic basis in salmonids. Conversely, the absence of a body mass effect in our experiment should not be interpreted as evidence that body size is irrelevant to hypoxia tolerance. Instead, the relationship between body size and hypoxia tolerance is still debated and appears to be strongly context-dependent. Comparative studies suggest that larger fish tend to be more sensitive to hypoxia in warmer waters, whereas body size often explains only a small proportion of the total variation in hypoxia responses [17,72]. Therefore, in the present study—conducted on relatively small, fasted parr with a limited body-weight range—the signal attributable to family- and individual-level variation may reasonably have outweighed any effect of body mass. The calculation of the k index, based on the length and weight of the fish, indicates that a value greater than 1.1 would suggest that the fish are in good condition and that observed differences are not related to malnutrition or deformities. The k index, or condition factor, is a simple, rapid, and non-invasive assessment tool that can be used in aquaculture as an indicator of welfare [74]. Importantly, the demonstration of heritable variation in hypoxia tolerance has clear consequences for selective breeding programs designed to improve the strength of farmed Atlantic salmon to environmental stressors [34]. Nevertheless, further studies are needed to identify the genomic regions and candidate genes underlying variation in hypoxia tolerance, which would ultimately facilitate the development of genomic selection tools for this trait.

The blood data indicate that acute hypoxia imposed a marked disturbance on both the cellular and biochemical compartments, with erythrocyte-related variables particularly affected. Our results showed that both hypoxia-tolerant and hypoxia-susceptible fish exhibited lower hematocrit and hemoglobin levels than normoxic controls, while susceptible fish additionally displayed the lowest RBC counts. Mechanistically, these changes may reflect the combined effects of severe physiological stressors acting on the blood compartment, including acid–base disturbance, osmotic imbalance, and possible membrane instability or toxicity-like injury associated with extreme oxygen restriction [75,76,77]. Another factor to consider is the genetic background of the fish. Sadler et al. [78] reported differences in hematocrit, hemoglobin, and RBC counts between diploid and triploid female Atlantic salmon after a stress event, with triploid fish showing the lowest values. In the present study, however, ploidy was not determined, and therefore its potential contribution to the observed variation cannot be excluded. Interestingly, the fact that MCV and MCHC remained unchanged suggests that the observed alterations in RBC, hematocrit, and hemoglobin were more likely attributable to changes in cell number. However, similar alterations in oxygen transport in both phenotypes do not account for the differences observed in TLOE. Nevertheless, the response to hypoxia is a complex process that involves physiological, behavioral, and molecular changes, in which fish that adjust their metabolism toward an anaerobic state may have a greater likelihood of survival. This suggests that differences among parr-stage fish may be present at the molecular level. Additionally, a behavior observed in the fish during the trial was the absence of movement or a quiescent state, which reduces metabolic expenditure and could contribute to a longer TLOE.

Leukocyte-related changes were more subtle, yet they still suggest that susceptible fish may have experienced greater systemic disturbance under acute hypoxia. Although total leukocyte and lymphocyte counts did not differ significantly among groups, both variables showed a clear downward trend in susceptible fish. This pattern corresponds to a growing body of evidence indicating that hypoxia may modulate immune status in salmonids, with the magnitude and direction of these effects depending on the tissue analyzed and the duration and severity of oxygen deprivation. A study conducted by Niklasson et al. [79] showed that a reduction in oxygen levels (50% DO) over 6 to 7 weeks affected the intestinal immune system, characterized by reduced expression of pro-inflammatory genes and increased neutrophil numbers in the distal intestine of Atlantic salmon. Another study analyzed the effects of chronic hypoxia (52% DO) combined with pathogen infection on the head kidney and intestine, with results depending on the tissue and the pathogenic stimulus used. Nevertheless, the overall trend suggests that prolonged exposure to hypoxia diminishes the fish’s immune response [80]. On the other hand, the work conducted by Rojas et al. [39], using fish subjected to chronic hypoxia (40% OD) for 6 weeks and subsequently infected with *Aeromonas salmonicida*, suggests that exposure to hypoxia has different effects on genes related to an innate immune response in the head kidney of Atlantic salmon. Interestingly, their results show a reduction in lymphocyte numbers in fish subjected to hypoxia. Thus, the present blood cell data fit within a wider physiological framework in which lowered oxygen availability affects not only oxygen carriage but also the maintenance of immune homeostasis.

One of the most relevant findings of this study was the marked increase in blood lactate levels in fish exposed to hypoxia. At first glance, the particularly high lactate concentrations observed in fish classified as tolerant may appear contradictory; however, this pattern could be related to a longer exposure time and a greater cumulative reliance on anaerobic metabolism sustained by these fish before reaching loss of equilibrium (LOE). This interpretation is consistent with the Pasteur effect, in which hypoxia stimulates glycolytic flux to compensate for the reduced ATP production generated by oxidative phosphorylation, and this mechanism depends on HIF-1α activity [81]. Furthermore, recent evidence suggests that lactate may modify lysine residues in mitochondrial proteins, thereby limiting ATP production through oxidative phosphorylation [82]. On the other hand, the observed levels may not accurately reflect the true situation, as these fish could possess mechanisms that facilitate the conversion of this toxic metabolite into innocuous compounds. In salmonids, studies conducted on *Oncorhynchus mykiss* suggest that such mechanisms could involve increased lactate transport via facilitated diffusion or its conversion to glucose (and subsequently to glycogen). The conversion of lactate to glucose would occur in muscle, unlike what happens in mammals (a process known as the Cori cycle), due to the low expression of the main lactate transporter in muscle (MCT4) [83]. Blood glucose concentrations were also significantly elevated in hypoxia-exposed fish. This result is consistent with previous studies reporting increased plasma glucose levels in multiple fish species under hypoxic conditions, including European perch (*Perca fluviatilis*), gilthead seabream (*Sparus aurata*), nile tilapia (*Oreochromis niloticus*), grass carp (*Ctenopharyngodon idella*) and Atlantic salmon (*Salmo salar*) [23,84,85,86,87]. The higher glucose levels observed in tolerant fish suggest a stronger or more sustained metabolic response to oxygen limitation, which may have contributed to their greater resistance to hypoxia. In contrast, total protein concentrations were reduced in both hypoxia groups relative to controls. This decline is consistent with major metabolic reprogramming, in which protein catabolism may provide key metabolic intermediates required for purine synthesis [88]. Finally, the increase in serum hemoglobin provides a plausible link between the cellular and biochemical findings, as it is compatible with erythrocyte disruption or hemoglobin release into the extracellular fraction. Nevertheless, this result should be interpreted with caution, since serum hemoglobin may also be influenced by pre-analytical factors, such as sampling [89]. An additional methodological limitation is that hematological and serum biochemical/qPCR analyses had to be performed on different fish. Given the small size of the parr and the limited blood volume that could be obtained from the caudal vein—likely further reduced under acute hypoxia, when fish exhibit hypoxic bradycardia [90]—the use of separate analytical subsets was methodologically justified and likely unavoidable. Despite these limitations, the hematological and biochemical data provide valuable insight into the systemic response of Atlantic salmon to acute hypoxia.

At the transcriptional level, the tolerant phenotype was characterized by higher *hif1α* expression, lower *phd3* expression, higher *k6pp* expression, a tendency toward higher *ldhb* expression. This pattern is biologically meaningful because HIF-1α is a central regulator of hypoxic adaptation, orchestrating the transcriptional response to oxygen deprivation by inducing genes involved in glycolysis, angiogenesis, erythropoiesis, and other adaptive pathways [91]. In this context, the downregulation of *phd3* in tolerant fish, and probably the lower levels of PHD3 protein, would be expected to promote HIF-1α stabilization and prolong the hypoxia-responsive transcriptional program [92]. The upregulation of *k6pp*, a key regulator of glycolytic flux [93], further supports the idea that tolerant fish mounted a more effective metabolic compensation response, characterized by enhanced glycolytic capacity. Although *ldhb* did not differ significantly at the transcriptional level, this may reflect the fact that HIF-1α activity is more commonly associated with the regulation of *ldha* than *ldhb* [94]. Together, these data suggest that the stronger molecular response observed in tolerant fish likely contributed to their superior hypoxia tolerance by promoting metabolic adaptation and helping maintain cellular energy homeostasis under severe oxygen limitation.

The increased expression of *sod* suggests that tolerant fish mounted a stronger antioxidant response under acute hypoxic stress, which may have contributed to their superior hypoxia tolerance. However, these findings are not conclusive and warrant further investigation. During hypoxia, reactive oxygen species (ROS) levels can rise markedly, promoting cellular stress and oxidative damage [95]. Superoxide dismutase is a key antioxidant enzyme that catalyzes the dismutation of superoxide radicals (O_2_^−^) into hydrogen peroxide (H_2_O_2_) and molecular oxygen, thereby protecting cells against oxidative injury. Recent evidence indicates that *sod* expression increases during hypoxia and reoxygenation in teleost fish [96], supporting the interpretation that enhanced antioxidant capacity is an important component of the tolerant phenotype.

An intriguing finding of this study was the differential expression of immune-related genes between tolerant and susceptible fish. Specifically, *il15* expression was higher in tolerant fish, whereas *anx1* expression was higher in susceptible fish. To date, there is no direct evidence linking interleukin-15 (IL-15) to acute hypoxia in fish. IL-15 is a cytokine expressed in many non-lymphoid tissues, including the liver and skeletal muscle. In mammals, this cytokine is thought to play an important role in energy production within muscle cells by increasing glucose transport [97]. A study conducted in mice (*Mus musculus*) showed that this cytokine may be involved in the proliferation and growth of resident intestinal lymphocytes, through a mechanism that involves nutrient uptake (including glucose) and increased activity of the electron transport chain [98]. This information could be of interest in a hypoxic context, as behavioral changes in fish may lead them to seek areas with higher oxygen levels, resulting in increased muscular activity, which in turn could be associated with greater expression of this cytokine. On the other hand, Annexin 1 is a phospholipid- and calcium-binding protein involved in multiple cellular processes, including the resolution of inflammation, membrane trafficking, and apoptosis [99]. A study conducted in *Larimichthys crocea* showed that hypoxia can induce apoptosis both in vitro and in vivo through intrinsic (caspase 3) and extrinsic mechanisms [100]. Interestingly, the apoptotic process mediated by annexin 1 would involve caspase 3 activity in immune cells [99]. Based on this information, it can be suggested that the increase in *anx1* expression could be related to a lower number of circulating lymphocytes.

Despite the findings obtained in our research, there are limitations that reduce the statistical power and generalizability of our data. On one hand, it is important to mention the effect of temperature, as the fish were subjected to a 5 °C increase over a short period. Scientific evidence suggests that temperature, together with hypoxia-induced stress, modulates hematocrit levels in *Salmo salar* [101]. However, that study was conducted under moderate hypoxia conditions (70%) and at a temperature close to 20 °C; furthermore, the fish analyzed were in seawater, making direct comparison between the two studies difficult. Nevertheless, it is important to note that this temperature change may have contributed to increased stress levels in the fish, and consequently, to elevated plasma glucose levels. This possibility is supported by the increase in blood glucose in the presence of epinephrine (a molecule secreted under stress) in *Oncorhynchus mykiss* [83]. An increase in circulating glucose may have facilitated lactate production or depleted its reserves when oxygen levels began to decrease. Another factor was the limited number of fish analyzed. Scientific articles indicate that, to detect minimal significant differences between groups, it is necessary to have more than 25 but fewer than 100 fish [102]. However, this value is suggested to reduce the minimum detectable difference (MDD), and it should be considered that, in many cases, the number of samples analyzed is related to resource availability. Nevertheless, it is very important to establish a balance between these elements, depending on the objectives of the study to be conducted. Finally, the results obtained are derived from a specific cohort of fish and particular rearing conditions. Therefore, it is important to conduct new experiments that modify these parameters, as well as experiments that replicate these conditions, in order to validate the results obtained and confirm that the observed phenomena are not due to chance findings [103]. Overall, the data obtained in the present study suggest that the tolerant phenotype is characterized by a more integrated metabolic response to hypoxia, in which enhanced glucose mobilization may support energy metabolism.

## 5. Conclusions

Our study indicates that acute hypoxia tolerance is a biologically robust and aquaculture-relevant phenotype in Atlantic salmon parr. The results showed that hypoxia-tolerant fish maintained equilibrium for a longer period and withstood lower dissolved oxygen concentrations before losing their ability to maintain equilibrium, which was associated with a transcriptional profile suggesting activation of the HIF signaling pathway. Furthermore, the findings indicated that tolerance varied among families, but this trait was not related to body weight. These results suggest that resilience to acute hypoxia is influenced more by inherited physiological capacity than by simple differences in size.

In addition, our data suggest that blood could serve as a non-lethal, integrative tissue for the future identification of hypoxia-associated phenotypes in teleost fish. The validation of blood biomarkers could constitute a powerful tool for identifying resilient fish, enabling improved health monitoring in aquaculture settings with the aim of enhancing the sustainability of Atlantic salmon under increasingly challenging environmental conditions.

## Figures and Tables

**Table 1:** Families included in the study and descriptive statistics for body weight and length. Values are presented as mean ± standard deviation (SD).

Family	Dam/Sire	Sample Size (n)	Weight (g)	Length (cm)
Fam08	Dam01/Sire01	16	68.1 ± 17.5	17.7 ± 1.2
Fam07	Dam03/Sire02	12	69.3 ± 19	18 ± 1.8
Fam06	Dam05/Sire03	13	43.7 ± 14.9	15.3 ± 1.9
Fam04	Dam07/Sire04	12	56.0 ± 16.8	16.4 ± 1.4
Fam09	Dam06/Sire03	20	45.4 ± 12.2	15.7 ± 1.3
Fam01	Dam02/Sire01	15	72.3 ± 20	18.1 ± 1.6
Fam03	Dam09/Sire05	13	38.8 ± 8	15 ± 1.1
Fam02	Dam10/Sire05	18	47.9 ± 8.8	15.9 ± 1.1
Fam05	Dam08/Sire04	16	66.9 ± 11.1	17.5 ± 0.9
Fam10	Dam04/Sire02	15	65.7 ± 11.4	17.5 ± 1.1
**Overall**		**150**	57.2 ± 18.2	16.7 ± 1.7

**Table 2:** Primers used in RT-qPCR. F and R: forward and reverse sequences, respectively. Primer efficiency (E). Accession number obtained from National Center of Biotechnology Information (NCBI).

Gene	Primer Sequence	E	Accession Number
*elf-1α* (elongation factor 1 alpha)	F: GCTTACAAAATCGGCGGTAT	2.02	NM_001141909.1
	R: CTTGACGGACACGTTCTTGA		
*hprt* (hypoxanthine-guanidine phosphoribosyl transferase)	F: CTGACATTGCCCCTCAGATT	1.99	XM_014212855.2
	R: CACGCCTCAGAAATGTTCAA		
*hif1α* (hypoxia-inducible factor, isoform 1)	F: AAGTTGTTTGCCGTCTGTCC	1.99	NM_001140022.1
	R: CACCAAGTGTCTCGGTCTCT		
*phd3* (prolyl hydroxylase, isoform 3)	F: GTGCTATCCAGGAAATGGG	2.06	XM_014194431.2
	R: TGCAACGTAGGATTTGCC		
*k6pp* (ATP-dependent 6-phosphofructokinase)	F: CTGGGAACAAAGAGAACCC	2.02	BT059256.1
	R: CAGCAAACACTCAAACGC		
*ldhb* (L-lactate dehydrogenase B)	F: ATCTAGACACACACCTACCC	1.98	BT045973.1
	R: ATGGAAACACAGCAACCC		
*ndua6* (NADH dehydrogenase [ubiquinone] 1 alpha subcomplex subunit 6)	F: GCTGCTAAAGTTGTTAAACCG	2.08	BT047007.1
	R: CCTCACTTTGTCTGTTCCC		
*sod* (superoxide dismutase)	F: CCACGTCCATGCCTTTGG	2.05	NM_001123587.1
	R: TCAGCTGCTGCAGTCACGTT		
*il15* (interleukin 15)	F: GGAGTTGGAGGTTGTCCTGA	1.90	NM_001279065.1
	R: ATTGGCGTTCATGTTCTTCC		
*anx1* (annexin 1)	F: ATGGTGAACCAGGAACTTGC	1.90	NM_001141271.1
	R: ATAGCCTTTGCCACATCCAC		

**Table 3:** Normalized TLOE values for families used in this study. The mean k index of the analyzed family is showed.

Family	N	Normalized TLOE Value	Confidence Interval (95%)	k Index
Fam01	15	1.122 ± 1.207	0.454 ± 1.791	1.2 ± 0.12
Fam02	18	0.702 ± 0.922	0.243 ± 1.160	1.2 ± 0.14
Fam03	13	−0.046 ± 0.837	−0.552 ± 0.460	1.2 ± 0.15
Fam04	12	−0.060 ± 0.848	−0.598 ± 0.479	1.2 ± 0.14
Fam05	16	−0.105 ± 0.931	−0.601 ± 0.391	1.2 ± 0.14
Fam06	13	−0.112 ± 0.911	−0.663 ± 0.438	1.2 ± 0.14
Fam07	12	−0.141 ± 0.667	−0.565 ± 0.283	1.2 ± 0.15
Fam08	16	−0.149 ± 0.800	−0.576 ± 0.277	1.2 ± 0.15
Fam09	20	−0.594 ± 0.833	−0.984 ± −0.204	1.2 ± 0.14
Fam10	15	−0.604 ± 0.454	−0.856 ± −0.353	1.2 ± 0.16

## Data Availability

The data presented in this study is available on request from the corresponding author.

## Supplementary Materials

The following supporting information can be downloaded at: https://www.mdpi.com/article/10.3390/ani16162582/s1. Figure S1. Dissolved oxygen profile during the progressive acute hypoxia challenge. Dissolved oxygen concentration (mg L^−1^) was monitored over time in four experimental tanks during the loss of equilibrium (LOE) assay. Arrows indicate the time points at which the first and last fish lost equilibrium in each tank. The interval between these events represents the time to loss of equilibrium (TLOE) range within each tank and reflects individual variation in hypoxia tolerance.

## Abbreviations

The following abbreviations are used in this manuscript:

HIF-1α	Hypoxia-inducible factor 1 alpha
LOE	Loss of equilibrium
TLOE	Time to loss of equilibrium
EDTA	Ethylenediaminetetraacetic acid
Hct	Hematocrit
Hgb	Hemoglobin
RBC	Red blood cells
MCV	Mean corpuscular volume
MCHC	Mean corpuscular hemoglobin concentration
WBC	White blood cells
LYM	Lymphocytes
MID	Mid-size cells
GRA	Granulocytes
phd3	Prolyl hydroxylase domain 3
k6pp	6-phosphofructo-2-kinase
ldhb	Lactate dehydrogenase b
ndua6	NADH dehydrogenase ubiquinone 1 alpha subcomplex subunit 6
sod	Superoxide dismutase
il15	Interleukin 15
anx1	Annexin 1
elf1α	Elongation factor 1 alpha
hprt	hypoxanthine-guanidine phosphoribosyl transferase
ROS	Reactive oxygen species
