# Supplementary material for: Blood-Based Characterization of Hypoxia-Tolerant and Hypoxia-Susceptible Atlantic Salmon (Salmo salar) Under Acute Hypoxic Stress
Source: Animals (Basel). 2026 Aug 18;16(16):2582. doi: 10.3390/ani16162582 (PMC13508933; doi:10.3390/ani16162582)
Supplement: Supplementary file 1 [file animals-16-02582-s001.zip › animals-4389549-supplementary.pdf]

Blood-Based Characterization of Hypoxia-Tolerant and Hypoxia-Susceptible Atlantic Salmon (*Salmo salar*) under Acute Hypoxic Stress

Salinas-Parra Nicolás [1]*, Caballero Paz [1,4], Romero Alfonso [2], Martínez Karin [3], Soto Carlos [2] and Gallardo-Matus José [1]

*Corresponding author: nicolas.salinas@pucv.cl

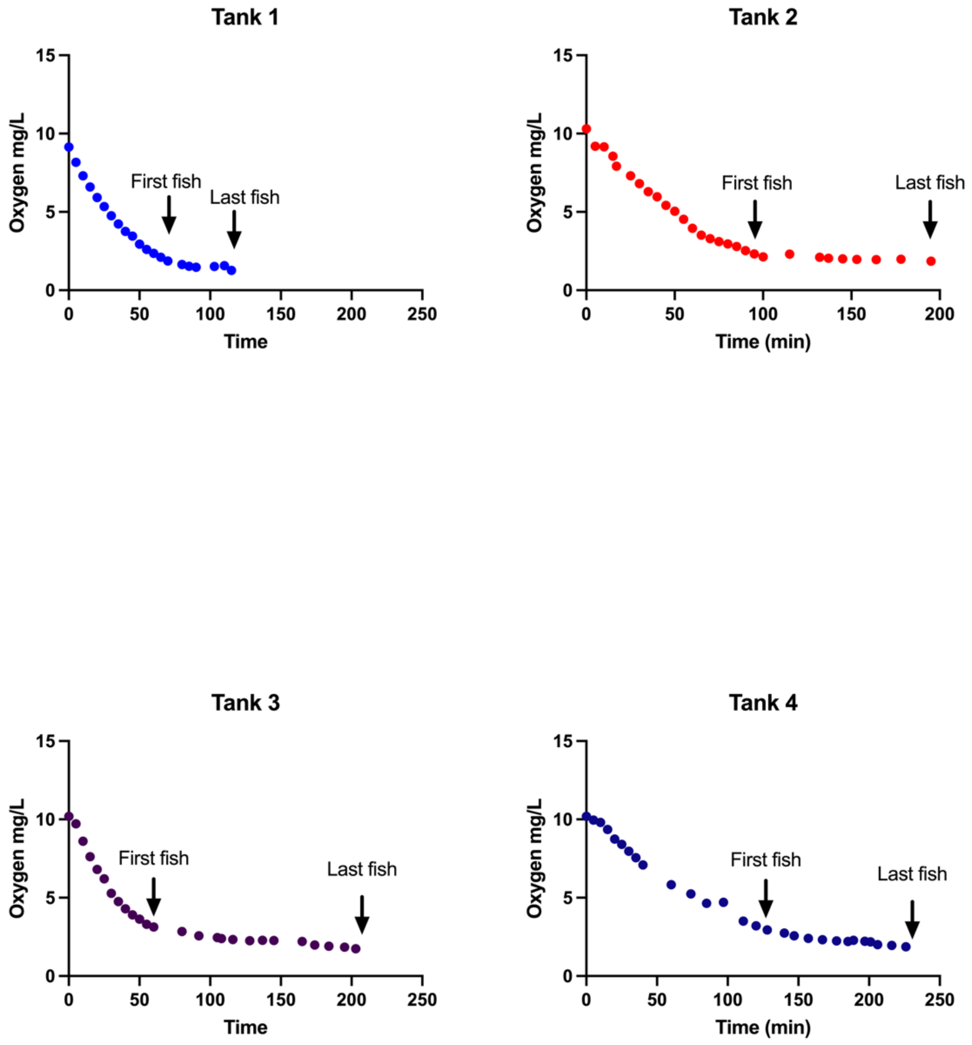

Figure S1. Dissolved oxygen profile during the progressive acute hypoxia challenge. Dissolved oxygen concentration (mg L^{-1}) was monitored over time in four experimental tanks during the loss of equilibrium (LOE) assay. Arrows indicate the time points at which the first and last fish lost equilibrium in each tank. The interval between these events represents the time to loss of equilibrium (TLOE) range within each tank and reflects individual variation in hypoxia tolerance.
